# Association of Thigh Thickness and Femoral Notch Width with Anterior Cruciate Ligament Attachment Size and Tear Risk

**DOI:** 10.3390/diagnostics16101531

**Published:** 2026-05-18

**Authors:** Waleed Albishi, Abdulrahman Alaseem, Mohammed N. Alhuqbani, Abdulmalik A. Alduraibi, Abdulaziz S. AlNahari, Eissa G. Bakri, Abdulmonem Alkhateeb, Faten Almohideb

**Affiliations:** 1 Department of Orthopedic Surgery, College of Medicine, King Saud University, Riyadh 11461, Saudi Arabia; 2 College of Medicine, King Saud University, Riyadh 11461, Saudi Arabia; abzsaad2017@gmail.com; 3 Department of Radiology, Jazan Health Cluster, Jazan 45142, Saudi Arabia; 4 Department of Radiology, Alahsa Health Cluster, Alahsa 36361, Saudi Arabia; 5 Department of Radiology, College of Medicine, King Saud University, Riyadh 11461, Saudi Arabia

**Keywords:** anterior cruciate ligament, ACL injury, femoral notch width, thigh thickness, MRI morphometry, ACL footprint, knee anatomy

## Abstract

**Background/Objectives**: An accurate understanding of anterior cruciate ligament (ACL) morphology is essential for individualized surgical planning in ACL reconstruction. Morphometric parameters of the knee, including the femoral notch width and surrounding soft tissue characteristics, may influence native ACL dimensions and potentially assist in preoperative graft sizing. **Methods**: This retrospective case–control study analyzed medical records, radiographs, and knee magnetic resonance imaging (MRI) performed at a tertiary academic medical center. Variables collected included femoral notch width, thigh thickness, and ACL attachment dimensions at the femoral and tibial insertions. Comparisons between patients with ACL tears and those with intact ACLs were also performed. Correlation analyses were performed to evaluate associations between morphometric parameters and ACL attachment size. Multivariable linear regression models were constructed to identify independent predictors after adjusting for age, sex, body mass index (BMI), limb side (left or right leg), and ACL status. **Results**: A total of 600 participants were included. The mean femoral notch width was 21.55 ± 6.15 mm, and the mean thigh thickness was 53.05 ± 11.66 mm. The mean ACL femoral and tibial attachment sizes were 8.12 ± 2.57 mm and 11.79 ± 3.89 mm, respectively. Thigh thickness demonstrated weak but significant positive correlations with both ACL femoral (r = 0.168, *p* = 0.001) and tibial attachment sizes (r = 0.236, *p* < 0.001). Femoral notch width showed a borderline association with ACL femoral attachment size (r = 0.092, *p* = 0.068) and a weak but significant correlation with ACL tibial attachment size (r = 0.095, *p* = 0.039). ACL tear group exhibited smaller thigh thickness measurements compared with controls (49.80 ± 12.00 mm vs. 55.65 ± 14.80 mm, *p* < 0.001) and smaller femoral notch width measurements compared with controls (21.20 ± 3.40 mm vs. 22.50 ± 3.18 mm, *p* = 0.001). Moreover, further analysis demonstrated that ACL tear status was associated with smaller measured ACL attachment sizes (*p* < 0.001). **Conclusions**: Thigh thickness and femoral notch width demonstrate measurable association with ACL attachment dimensions and differ between patients with ACL tears and those with intact ligaments. These findings indicate that both osseous and soft-tissue morphometric characteristics may influence ACL morphology and susceptibility to injury. Comprehensive preoperative imaging assessment of these anatomical parameters may help to optimize individualized surgical planning in ACL reconstruction.

## 1. Introduction

Anterior cruciate ligament (ACL) injuries are among the most common ligamentous injuries of the knee, with a particularly high incidence in pivoting sports such as football, basketball, and volleyball. Such injuries are most common among young, physically active individuals aged 14–25 years [1]. Rupture of the ACL results in significant functional impairment due to the loss of the ligament’s primary role in restraining anterior tibial translation and controlling the rotational stability of the knee, predisposing the joint to recurrent instability, secondary meniscal injury, and progressive cartilage degeneration [2]. A recent systematic review reported a sevenfold increase in the risk of knee osteoarthritis following ACL injury, with approximately 36% of patients who undergo reconstruction developing osteoarthritis within 10 years [2]. Anatomically, the ACL consists of two functional bundles—the anteromedial and posterolateral bundles—that work synergistically to maintain stability throughout the knee’s range of motion. Consequently, a comprehensive understanding of ACL anatomy remains essential for optimizing reconstruction strategies and improving long-term clinical outcomes [3].

There is considerable interindividual variability in the dimensions of the ACL, influenced by factors such as sex, body height, and ethnicity, which directly affect the native footprint size and the surgical approach to reconstruction [4]. Recent three-dimensional MRI-based investigations have demonstrated significant race- and sex-specific differences in the femoral and tibial ACL footprint locations, which highlights that a uniform approach to tunnel placement may be inadequate [5]. This anatomical variability is particularly relevant during graft selection, as the graft should approximate the native ACL dimensions to restore physiological knee kinematics. Studies have shown that hamstring autografts with a diameter of less than 8 mm are associated with higher graft failure rates, emphasizing the importance of preoperative estimation of ACL size [6]. Accordingly, the concept of anatomic ACL reconstruction has evolved to emphasize individualized restoration of the native insertion sites, bundle orientation, and graft dimensions with the goals of replicating native knee biomechanics and improving clinical outcomes [7].

In addition to ligament morphology, the osseous anatomy of the distal femur—particularly the intercondylar notch—has increasingly been recognized as an important anatomical factor associated with ACL injury risk [8]. The notch width index (NWI), defined as the ratio of the intercondylar notch width to the bicondylar width of the distal femur, as measured on MRI, has been widely used to quantify notch stenosis, with lower values indicating a narrower notch and a potentially increased risk of ACL rupture [9]. A recent systematic review has confirmed that a narrow intercondylar notch is consistently associated with a higher risk of primary ACL injury and may also contribute to graft failure following reconstruction due to mechanical impingement and constrained tunnel placement [8]. These morphometric assessments, together with other structural parameters such as notch shape and posterior tibial slope, highlight the importance of detailed preoperative imaging evaluation to support individualized surgical planning and improve reconstruction outcomes [10].

Beyond femoral notch morphology, several studies have explored whether anthropometric and morphometric parameters can help estimate native ACL dimensions or support preoperative graft planning [11]. However, the available evidence remains limited and heterogeneous. Prior studies have shown that anthropometric variables such as height, weight, and thigh circumference may correlate with hamstring graft size, but the strength and consistency of these associations vary across studies and populations [11]. Likewise, MRI-based studies have suggested that certain patient-level and knee morphometric measures may help predict ACL-related dimensions, although direct evidence for soft-tissue surrogates such as thigh thickness remains sparse [12,13,14]. In contrast, research on ACL injury risk has focused predominantly on osseous and knee geometric factors, particularly intercondylar notch morphology, notch width index, and tibial slope [8,10,15]. In the present study, femoral notch width was selected as a directly measured osseous parameter to evaluate its association with ACL attachment size alongside thigh thickness; however, other established notch-related metrics such as notch width index and notch angle were not included in the prespecified measurement set and should be considered in future studies [16]. Accordingly, whether thigh thickness and absolute femoral notch width are meaningfully associated with native ACL attachment size or ACL tear status remains insufficiently defined. Therefore, the present study evaluated the association between femoral notch width, thigh thickness, and ACL attachment size using MRI and investigated whether these morphometric parameters differ between patients with torn ACLs and those with intact ACLs.

## 2. Materials and Methods

### 2.1. Study Design and Setting

This retrospective cohort study was conducted at a tertiary academic medical center in Riyadh, Saudi Arabia. The study was performed in collaboration with the Departments of Orthopedic Surgery and Radiology. Institutional review board approval was obtained prior to the initiation of the study (IRB approval number: No. E-23-7383, 5 October 2023).

### 2.2. Study Population

Knee magnetic resonance imaging (MRI) examinations performed between January 2015 and December 2025 were screened for eligibility. Adult patients aged 18–45 years were included if MRI quality was sufficient for accurate morphometric assessment. Participants were categorized according to ACL status on MRI into a torn-ACL group and an intact-ACL control group. ACL tears were identified based on MRI findings; however, injury mechanism was not included, because these data were not consistently available in a standardized manner across all retrospective cases.

Patients were excluded if they had concomitant ligamentous injury (including posterior cruciate or collateral ligament injuries), meniscal tears, previous knee surgery, previous knee fracture, poor-quality MRI, or imaging outside the predefined study period. The control group was derived from patients undergoing knee MRI during primary clinical care who demonstrated an intact ACL and no exclusionary structural abnormalities; therefore, these controls were not necessarily asymptomatic healthy individuals. Radiographs were additionally reviewed to assess coronal lower-limb alignment and tibial plateau slope, and knees with significant coronal malalignment or increased tibial slope were excluded to reduce biomechanical confounding. When both knees of the same patient were available, one knee was randomly retained and the contralateral knee was excluded to avoid clustering.

### 2.3. MRI Acquisition Protocol

All MRI examinations were performed with the patient in the supine position with the knee in full extension using a dedicated knee coil. Non-enhanced MRI sequences were obtained according to the institutional imaging protocol. The standard protocol included sagittal T1 turbo spin-echo (TSE) without fat saturation, sagittal oblique T2 sequences without fat saturation, sagittal proton-density (PD) TSE sequences with fat saturation, coronal PD TSE with fat saturation, and axial PD TSE with fat saturation. Slice thickness ranged between approximately 2.5 and 3.5 mm, depending on the sequence. Images were reviewed using the hospital’s Picture Archiving and Communication System (PACS), which was also used for all morphometric measurements.

### 2.4. Morphometric Measurements

All morphometric measurements were performed using standardized measurement tools within the Picture Archiving and Communication System (PACS). ACL measurements were obtained on sagittal oblique T2-weighted sequences without fat saturation. ACL width was assessed at the femoral attachment and tibial attachment, and ACL length was measured as the linear distance between the femoral and tibial attachment sites.

Femoral notch morphology was assessed on axial proton-density fat-suppressed sequences. Maximum femoral notch width was measured at the midpoint of the intercondylar notch in the axial plane, with localization confirmed on the corresponding coronal images.

Thigh thickness was measured on lateral knee radiographs obtained with the knee flexed. The measurement was defined as the maximum soft-tissue thickness measured perpendicular to the anterior femoral cortex at a point 10 cm proximal to the superior-posterior border of the patella. This parameter was used as a standardized radiographic representation of regional thigh soft-tissue bulk rather than a direct measure of isolated muscle mass.

All MRI and radiographic measurements were independently obtained by two musculoskeletal radiologists using standardized measurement protocols.

### 2.5. Variables Collected

Demographic and anthropometric variables collected for analysis were age, sex, height, weight, body mass index (BMI), and limb side (right or left leg). Morphometric parameters included femoral notch width, thigh thickness, ACL femoral attachment width, and ACL tibial attachment width. ACL status (torn vs. intact) was recorded based on MRI findings.

### 2.6. Statistical Analysis

All statistical analyses were performed using IBM SPSS Statistics version 21 (IBM Corp., Armonk, NY, USA). Continuous variables were assessed for normality using the Shapiro–Wilk test and were expressed as mean ± standard deviation (SD). Correlations between femoral notch width, thigh thickness, and ACL attachment size were evaluated using Pearson’s correlation coefficient with corresponding *p*-values.

Multivariable linear regression analyses were performed to determine independent predictors of ACL attachment size. Predictor variables were selected a priori based on the study objectives and their potential clinical relevance. Femoral notch width and thigh thickness were entered as the primary explanatory variables, while age, sex, BMI, limb side, and ACL status were included as prespecified covariates to account for potential confounding. Collinearity diagnostics were evaluated using variance inflation factors (VIFs). A two-sided *p*-value < 0.05 was considered statistically significant.

### 2.7. Use of Artificial Intelligence (AI) Tools

During the preparation of this manuscript, the authors used ChatGPT (GPT-5.4 Thinking, OpenAI) for text editing, including grammar, structure, spelling, punctuation, and paragraph formatting. The authors reviewed and edited the output and take full responsibility for the content of this publication.

## 3. Results

### 3.1. Baseline Characteristics of the Study Cohort

A total of 600 participants were included in the analysis. The mean age of the cohort was 33.26 ± 7.98 years, with a mean body mass index (BMI) of 27.56 ± 5.23 kg/m^2^. The study population consisted predominantly of males (460, 76.7%), while 140 participants (23.3%) were female. Regarding limb distribution, 335 knees (55.8%) were right-sided, and 265 (44.2%) were left-sided.

Based on MRI findings, 293 knees (48.8%) had intact ACLs and were classified as controls, whereas 307 knees (51.2%) demonstrated complete ACL tears. Over the whole cohort, the mean femoral notch width was 21.55 ± 6.15 mm, and the mean thigh thickness measured was 53.05 ± 11.66 mm. The mean ACL femoral attachment size was 8.12 ± 2.57 mm, while the mean ACL tibial attachment size was 11.79 ± 3.89 mm.

A detailed summary of the demographic and morphometric characteristics of the study population is presented in Table 1.

### 3.2. Comparison of Morphometric Parameters Between ACL Tear and Control Groups

Comparisons of morphometric parameters between patients with ACL tears and those with intact ACLs demonstrated significant differences in selected measurements. Patients with ACL tears exhibited smaller thigh thickness measurements compared with controls (49.80 ± 12.00 mm vs. 55.65 ± 14.80 mm, *p* < 0.001). Moreover, further analysis demonstrated that the ACL tear group had smaller femoral notch width measurements compared with controls (21.20 ± 3.40 mm vs. 22.50 ± 3.18 mm, *p* = 0.001). These findings suggest that both soft-tissue and osseous morphometric characteristics may be associated with ACL injury status.

### 3.3. Correlation Between Morphometric Parameters and ACL Attachment Size

Correlation analyses were performed to evaluate the relationship between femoral notch width, thigh thickness, and ACL attachment dimensions.

Femoral notch width demonstrated a weak, non-significant correlation with ACL femoral attachment size (r = 0.092, *p* = 0.068). However, a weak but statistically significant positive correlation was observed with ACL tibial attachment size (r = 0.095, *p* = 0.039).

Thigh thickness demonstrated weak but statistically significant positive correlations with both ACL attachment measurements. Specifically, thigh thickness correlated with ACL femoral attachment size (r = 0.168, *p* = 0.001) and ACL tibial attachment size (r = 0.236, *p* < 0.001).

These correlation findings are summarized in Table 2.

### 3.4. Multivariable Linear Regression Analysis for ACL Femoral Attachment Size

A multivariable linear regression analysis was conducted to identify independent predictors of ACL femoral attachment size, adjusting for age, sex, BMI, limb side, and ACL status.

In the adjusted model, sex was independently associated with ACL femoral attachment size, with males demonstrating larger femoral attachment measurements (B = 0.804, β = 0.155, 95% CI 0.213–1.394, *p* = 0.008). Moreover, ACL status showed a significant association, with ACL tear status associated with smaller measured femoral attachment size (B = −1.183, β = −0.212, 95% CI −1.832 to −0.534, *p* < 0.001).

Femoral notch width (*p* = 0.166), thigh thickness (*p* = 0.079), age (*p* = 0.177), BMI (*p* = 0.613), and limb side (*p* = 0.380) were not independently associated with ACL femoral attachment size after adjustment.

Full results of the multivariable regression model for ACL femoral attachment size are presented in Table 3.

### 3.5. Multivariable Linear Regression Analysis for ACL Tibial Attachment Size

A second multivariable regression model was constructed to evaluate independent predictors of ACL tibial attachment size.

In this model, femoral notch width remained independently associated with ACL tibial attachment size (B = 0.047, β = 0.087, 95% CI 0.006–0.088, *p* = 0.024). Sex was also independently associated with tibial attachment size, with males demonstrating larger measurements (B = 1.210, β = 0.143, 95% CI 0.466–1.953, *p* = 0.001). Furthermore, ACL tear status demonstrated a strong negative association with tibial attachment size (B = −5.185, β = −0.655, 95% CI −5.895 to −4.476, *p* < 0.001).

Thigh thickness (*p* = 0.326), age (*p* = 0.458), BMI (*p* = 0.312), and limb side (*p* = 0.494) were not significantly associated with ACL tibial attachment size in the adjusted model.

Detailed regression results are presented in Table 4.

### 3.6. Graphical Representation of Morphometric Associations

**Figure 1:**
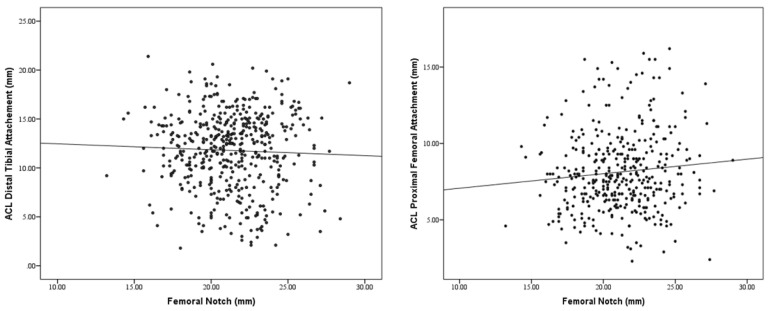
Scatter Plots Displaying the Association Between Femoral Notch Width and ACL Attachment Size.

**Figure 2:**
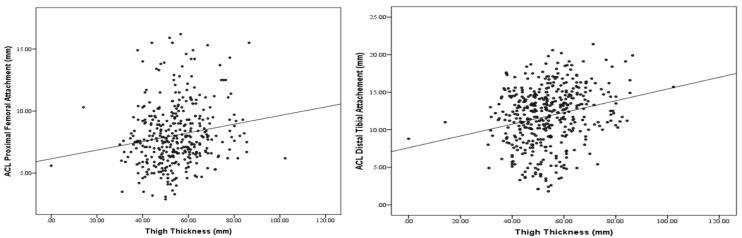
Scatter Plots Displaying the Association Between Thigh Thickness and ACL Attachment Size.

Scatter plots illustrating the relationships between femoral notch width and ACL attachment size and between thigh thickness and ACL attachment size are shown in Figure 1 and Figure 2, respectively.

## 4. Discussion

In this MRI-based morphometric study, we found that patients with ACL tears had smaller thigh thickness and smaller femoral notch width measurements than those with intact ACLs. Correlation analyses showed weak associations between the evaluated morphometric parameters and ACL attachment size: thigh thickness demonstrated weak positive correlations with both femoral and tibial ACL attachment measurements, whereas femoral notch width showed a weak association only with ACL tibial attachment size and no significant association with ACL femoral attachment size. In the adjusted regression models, femoral notch width remained independently associated with ACL tibial attachment size, while male sex was independently associated with larger femoral and tibial ACL attachment dimensions. Overall, these findings suggest that selected osseous and soft-tissue morphometric characteristics are related to ACL morphology, although the strength of these associations appears limited.

The morphology of the femoral intercondylar notch has long been recognized as an important anatomical factor associated with ACL injury risk. MRI-based investigations have demonstrated that a narrower intercondylar notch width and a lower notch width index are significantly associated with an increased risk of ACL rupture, likely due to reduced space for the ligament and potential mechanical impingement during dynamic knee loading [17]. Notch morphology has been reported to affect the size of the ACL and to influence the risk of injury. Cadaveric and MRI-based morphometric studies have demonstrated positive correlations between intercondylar notch width and ACL cross-sectional area or volume, suggesting that individuals with a narrower notch may have a smaller ligament with reduced structural capacity [18]. Furthermore, a recent systematic review in skeletally immature patients has confirmed that a smaller notch width index represents a consistent morphological risk factor for ACL injury across age groups [19]. The findings of the present study partially support these observations. Although femoral notch width was not independently associated with ACL femoral attachment size after adjustment for confounding variables, a weak but statistically significant association was observed with ACL tibial attachment size. This finding suggests that, while notch morphology may reflect broader structural characteristics of the knee joint, its relationship with ACL dimensions may not be uniform across all attachment sites. However, the magnitude of these correlations was small, indicating that although statistically detectable, these linear associations are weak and should not be interpreted as strong standalone predictors of ACL attachment dimensions in clinical practice.

In addition to evaluating associations with ACL attachment size, our study also explored morphometric differences between patients with ACL tears and controls. Patients with ACL tears demonstrated significantly smaller thigh thickness compared with individuals with intact ACLs. This finding may be indicative of reduced muscular soft-tissue bulk surrounding the knee, which could influence the dynamic stabilization capacity of the joint during high-demand movements. Muscular structures around the knee play an important role in controlling rotational forces and anterior tibial translation, and adequate quadriceps strength has been shown to contribute to knee joint stability during functional activities [20]. However, this finding should be interpreted cautiously, as reduced thigh thickness in the ACL tear group may partly reflect post-injury muscle atrophy rather than a pre-injury anatomical predisposition. Regarding osseous morphology, an analysis demonstrated that patients with ACL tears had smaller femoral notch width measurements compared with controls. This observation is consistent with the concept that a narrower intercondylar notch may predispose the ligament to mechanical impingement during knee motion, thereby increasing the risk of ACL rupture. Together, these findings suggest that both soft-tissue and osseous anatomical characteristics may contribute to ACL injury susceptibility, although their influence may vary across different patient subgroups.

Several studies have attempted to identify reliable anthropometric predictors of ACL and graft dimensions to facilitate individualized surgical planning. A large systematic review and meta-analysis including 7110 patients has demonstrated that patient height exhibited the strongest positive correlation with hamstring autograft diameter, followed by body weight and thigh circumference, whereas age and sex were not consistently associated with graft size [11]. More recent MRI-based studies have extended these observations by investigating predictors of native ACL footprint morphology. For example, patient height has been shown to account for as much as 46% of the differences in ACL tibial footprint length. This difference helps surgeons to choose between single- and double-bundle reconstruction methods [13]. In addition, combining anthropometric measures with MRI-derived morphometric parameters such as femoral bicondylar width has been shown to improve the accuracy of preoperative ACL footprint prediction [14]. In the present study, thigh thickness demonstrated weak but statistically significant correlations with both femoral and tibial ACL attachment sizes in an unadjusted analysis. However, this association did not remain significant after adjustment for demographic and clinical covariates in multivariable models. These findings suggest that, while general body morphology may reflect broader anatomical characteristics of the knee, anthropometric measures alone may have limited independent predictive value for ACL attachment dimensions. Future studies may benefit from directly comparing stable anthropometric variables such as height with thigh thickness to determine which parameters are more informative for ACL morphometric assessment.

Sex-based differences in ACL morphology have consistently been reported in the literature and are considered an important factor contributing to the higher incidence of ACL injuries observed in females. MRI-based morphometric studies have demonstrated that females have significantly smaller ACL dimensions compared with males, including reduced ligament volume, cross-sectional area, and overall length, with these differences persisting even after adjustment for body size and bicondylar width [21]. Furthermore, the determinants of ACL size appear to vary between sexes. In males, the ACL cross-sectional area has been shown to correlate primarily with anthropometric parameters such as height and weight, whereas, in females, the area appears to be more strongly associated with intercondylar notch morphology, which is suggestive of sex-specific anatomical and biomechanical determinants [22]. The findings of the present study are consistent with these observations. In our multivariable regression models, male sex was independently associated with larger ACL femoral and tibial attachment dimensions, highlighting the substantial influence of sex on ligament morphology. These results support the concept that sex-specific anatomical differences should be considered during preoperative evaluation and surgical planning to ensure anatomically appropriate graft sizing and individualized ACL reconstruction strategies.

Accurate preoperative estimation of graft size is an important component of modern ACL reconstruction, as undersized grafts have been associated with inferior biomechanical properties and higher failure rates. Previous studies have demonstrated that hamstring autografts with a diameter smaller than 8 mm are linked to increased graft failure, whereas graft diameters of 9 mm or greater appear to provide improved biomechanical stability and lower re-rupture risk, particularly in revision settings [23]. Because the intraoperative graft diameter remains difficult to predict, several approaches have been proposed to improve preoperative planning, including the use of anthropometric parameters and MRI-based morphometric models [6]. More recently, advanced imaging techniques, such as three-dimensional MRI segmentation of the hamstring tendon cross-sectional area, have demonstrated promising accuracy for predicting quadrupled graft diameter prior to surgery [24]. Similarly, MRI-based protocols designed to identify patient-specific ACL footprint locations have reinforced the shift toward individualized anatomic reconstruction strategies rather than a standardized surgical approach [25]. The findings of the present study contribute to this evolving concept by demonstrating measurable associations between knee morphometric characteristics and ACL attachment dimensions. From a clinical perspective, these morphometric relationships may assist surgeons in anticipating ACL footprint size and optimizing graft selection during reconstruction, ultimately leading to improved surgical outcomes and patient recovery times.

### Strengths and Limitations

The present study has several important strengths. First, it involved a relatively large cohort of MRI examinations, allowing a comprehensive evaluation of morphometric relationships between femoral notch width, thigh thickness, and ACL attachment dimensions. Second, the study utilized standardized MRI-based measurement techniques, which provide high spatial resolution and allow accurate assessment of ligamentous and osseous structures of the knee. Third, by incorporating both osseous (femoral notch width) and soft tissue–related (thigh thickness) parameters in multivariable regression models, the study provides a more comprehensive evaluation of anatomical factors that may influence ACL attachment morphology. Finally, this study contributes population-specific morphometric data from a Middle Eastern cohort, a population that remains underrepresented in the literature on ACL anatomy and reconstruction planning.

Some limitations should be considered when interpreting these findings. The retrospective design introduces potential selection bias and precludes causal inference. Although the sample size was relatively large, the cohort was derived from a single-center local clinical population, and multicenter studies in more diverse populations are needed to confirm generalizability. In addition, the predominantly male distribution may further limit applicability to female patients despite adjustment for sex in the multivariable models. Thigh thickness was measured on lateral radiographs rather than MRI-based soft-tissue assessment, which may have provided more detailed anatomical characterization. Radiograph may reflect the combined effects of muscle mass, adipose tissue, body habitus, and post-injury change. Moreover, the interval between ACL injury and MRI acquisition was not consistently available in the ACL tear group, and post-injury disuse atrophy may therefore have contributed to the smaller thigh thickness observed in the group with torn ACLs. Finally, the control group consisted of patients with intact ACLs identified from routine clinical MRI examinations rather than asymptomatic healthy volunteers. Although strict exclusion criteria were applied to reduce structural confounding, selection bias remains possible, and the morphometric values in this group may not fully reflect those of a truly asymptomatic population.

## 5. Conclusions

In this MRI-based morphometric study, femoral notch width and thigh thickness showed weak but measurable associations with ACL attachment dimensions and differed between patients with torn ACLs and those with intact ligaments. These findings suggest that osseous and soft-tissue morphometric characteristics may be related to ACL morphology and ACL status, although their isolated clinical predictive value appears limited. Femoral notch width remained independently associated with ACL tibial attachment size, while male sex was a consistent predictor of larger ACL attachment dimensions. These results support a complementary, rather than standalone, role for morphometric assessment in preoperative evaluation and warrant further study before routine clinical application.

## Figures and Tables

**Table 1:** Baseline Demographic and Clinical Characteristics of the Study Cohort.

Variable	Overall Cohort Mean (SD)/ Frequency (%)	ACL Tear	Intact ACL	*p*-Value
		N-307 (51.2%)	N-293 (48.8%)	
Age (years)	33.26 (7.98)	32.60 (7.11)	33.95 (8.76)	0.040
BMI (kg/m^2^)	27.56 (5.23)	27.11 (4.82)	28.05 (5.63)	0.032
Height (cm)	170.33 (8.66)	172.96 (6.84)	167.36 (9.51)	<0.001
Weight (kg)	80.09 (17.11)	81.13 (15.29)	78.92 (18.92)	0.125
Sex—Male (%)	460 (76.7)	302 (98.4)	158 (53.9)	0.01
Sex—Female (%)	140 (23.3)	5 (1.6)	135 (46.1)	
Limb Side—Right (%)	335 (55.8)	187 (60.9)	148 (50.5)	0.01
Limb Side—Left (%)	265 (44.2)	120 (39.1)	145 (49.5)	
Femoral notch width (mm)	21.55 (6.15)	21.61 (2.25)	21.10 (2.69)	0.047
Thigh thickness (mm)	53.05 (11.66)	49.87 (10.37)	56.41 (12.02)	<0.001
ACL femoral attachment (mm)	8.12 (2.57)	7.17 (2.60)	8.48 (2.48)	<0.001
ACL tibial attachment (mm)	11.79 (3.89)	8.73 (3.44)	13.66 (2.82)	<0.001

**Table 2:** Correlation Between Morphometric Parameters and ACL Attachment Size.

Variable	ACL Femoral Attachment r, *p*	ACL Tibial Attachment r, *p*
Femoral notch width	0.092, 0.068	0.095, 0.039
Thigh thickness	0.168, 0.001	0.236, <0.001

**Table 3:** Multivariable Linear Regression Identifying Independent Predictors of ACL Femoral Attachment Size.

Variable	B (Unstandardized)	Standardized β	95% CI	*p*-Value
Femoral notch width (mm)	0.023	0.072	−0.010–0.056	0.166
Thigh thickness (mm)	0.023	0.113	−0.003–0.049	0.079
Age	0.021	0.073	−0.010–0.052	0.177
Sex	0.804	0.155	0.213–1.394	0.008
BMI	0.015	0.032	−0.042–0.071	0.613
Limb Side	−0.229	−0.046	−0.740–0.282	0.380
ACL Status	−1.183	−0.212	−1.832–−0.534	<0.001

**Table 4:** Multivariable Linear Regression Identifying Independent Predictors of ACL Tibial Attachment Size.

Variable	B (Unstandardized)	Standardized β	95% CI	*p*-Value
Femoral notch width (mm)	0.047	0.087	0.006–0.088	0.024
Thigh thickness (mm)	0.015	0.047	−0.015–0.046	0.326
Age	0.014	0.029	−0.022–0.049	0.458
Sex	1.210	0.143	0.466–1.953	0.001
BMI	0.034	0.048	−0.032–0.101	0.312
Limb Side	−0.203	−0.026	−0.788–0.381	0.494
ACL Status	−5.185	−0.655	−5.895–−4.476	<0.001

## Data Availability

The data presented in this study are available on request from the corresponding author due to privacy.

## Abbreviations

The following abbreviations are used in this manuscript:

ACL	Anterior Cruciate Ligament
MRI	Magnetic Resonance Imaging
BMI	Body Mass Index
PD	Proton Density
TSE	Turbo Spin-Echo
PACS	Picture Archiving and Communication System
IRB	Institutional Review Board
NWI	Notch Width Index
CI	Confidence Interval
VIF	Variance Inflation Factor
SPSS	Statistical Package for the Social Sciences
